# Maternal mortality decline in Zimbabwe, 2007/2008 to 2018/2019: findings from mortality surveys using civil registration, vital statistics and health system data

**DOI:** 10.1136/bmjgh-2022-009465

**Published:** 2022-08-04

**Authors:** Reuben Musarandega, Jenny Cresswell, Thulani Magwali, Davidzoyashe Makosa, Rhoderick Machekano, Solwayo Ngwenya, Lennarth Nystrom, Robert Pattinson, Stephen Munjanja, the Zimbabwe Maternal and Perinatal Mortality Study (ZMPMS) Group, Gwendoline Kandawasvika

**Affiliations:** 1 School of Health Systems and Public Health, University of Pretoria Faculty of Health Sciences, Pretoria, South Africa; 2 Unit of Obstetrics and Gynaecology, Faculty of Medical and Health Sciences, University of Zimbabwe, Harare, Zimbabwe; 3 Department of Sexual and Reproductive Health and Research, WHO, Geneve, Switzerland; 4 Department of Reproductive Health (fomerly), Ministry of Health and Child Care, Harare, Zimbabwe; 5 Department of Biostatistics and Epidemiology, Stellenbosch University, Cape Town, South Africa; 6 Department of Obstetrics and Gynaecology, National University of Science and Technology, Bulawayo, Zimbabwe; 7 Department of Epidemiology and Global Health, Umea University, Umea, Sweden; 8 Research Centre for Maternal, Fetal, Newborn & Child Health Care Strategies, University of Pretoria, Pretoria, South Africa

**Keywords:** epidemiology, health services research, maternal health, public Health

## Abstract

**Background:**

Sustainable Development Goal (SDG) 3.1 target is to reduce the global maternal mortality ratio (MMR) to less than 70 maternal deaths per 100 000 live births by 2030. In the Ending Preventable Maternal Mortality strategy, a supplementary target was added, that no country has an MMR above 140 by 2030. We conducted two cross-sectional reproductive age mortality surveys to analyse changes in Zimbabwe’s MMR between 2007–2008 and 2018–2019 towards the SDG target.

**Methods:**

We collected data from civil registration, vital statistics and medical records on deaths of women of reproductive ages (WRAs), including maternal deaths from 11 districts, randomly selected from each province (n=10) using cluster sampling. We calculated weighted mortality rates and MMRs using negative binomial models, with 95% CIs, performed a one-way analysis of variance of the MMRs and calculated the annual average reduction rate (ARR) for the MMR.

**Results:**

In 2007–2008 we identified 6188 deaths of WRAs, 325 pregnancy-related deaths and 296 maternal deaths, and in 2018–2019, 1856, 137 and 130, respectively. The reproductive age mortality rate, weighted by district, declined from 11 to 3 deaths per 1000 women. The MMR (95% CI) declined from 657 (485 to 829) to 217 (164 to 269) deaths per 100 000 live births at an annual ARR of 10.1%.

**Conclusions:**

Zimbabwe’s MMR declined by an annual ARR of 10.1%, against a target of 10.2%, alongside declining reproductive age mortality. Zimbabwe should continue scaling up interventions against direct maternal mortality causes to achieve the SDG 3.1 target by 2030.

**WHAT IS ALREADY KNOWN ON THIS TOPIC**

- Zimbabwe’s maternal mortality ratio (MMR) was last estimated at 462 maternal deaths per 100 000 live births against the Sustainable Development Goal (SDG) supplementary target for individual countries of an MMR no more than 140 by 2030.

**WHAT THIS STUDY ADDS**

- Zimbabwe’s MMR declined between 2007–2008 and 2018–2019 from 657 (95% CI: 485 to 829) to 217 (95% CI: 164 to 269) deaths per 100 000 live births at an annual average reduction rate (ARR) of 10.1% against an annual ARR of 10.2% needed to achieve the 2030 SDG target.

**HOW THIS STUDY MIGHT AFFECT RESEARCH, PRACTICE OR POLICY**

- This decline in Zimbabwe’s MMR was supported by temporal stability in the economy, substantial external funding and HIV treatment which has been optimised, hence Zimbabwe needs sustained maternal health funding to continue scaling up interventions against the direct causes of maternal mortality and regular assessment of the MMR, to achieve the SDG 3.1 target.

## Background

Reducing maternal mortality is a global priority, which is promoted by the Sustainable Development Goals (SDGs).^1–3^ The SDG 3.1 target is to achieve a global average maternal mortality ratio (MMR) of less than 70 maternal deaths per 100 000 live births by 2030. This global target requires every country to calculate and achieve its national target by 2030. Countries calculate their targets using the annual average reduction rate (ARR) needed to reduce the global average MMR to 70 and the country’s 2015 MMR as a baseline. High-burden countries whose targets remain high are to reduce their MMRs to no more than 140 per 100 000 by 2030.^1 4 5^

Zimbabwe is a sub-Saharan Africa lower-middle-income country with an estimated population of 15 million, a gross national income per capita of US$1100 in 2020 and a high MMR estimated at 462 maternal deaths per 100 000 live births in 2019.^6–9^ In 2007–2008, Zimbabwe was experiencing a socioeconomic crisis that started in the late 1990s,^10–12^ and resulted in severe hyperinflation and near-collapse of the country’s economic and social sectors.^10 11 13–15^ The gross domestic product shrunk from an annual growth rate of +10.4% in 2000 to −17.7% in 2008.^16^ Amid these economic challenges, significant improvements occurred in maternal, neonatal and child health (MNCH) and other health interventions—HIV, malaria and tuberculosis, with potential impact on maternal mortality (online supplemental table S1).

10.1136/bmjgh-2022-009465.supp1 Supplementary data

To analyse the epidemiology of maternal mortality in Zimbabwe we conducted two cross-sectional Reproductive Age Mortality Surveys (RAMOS) in 2007–2008 and 2018–2019, assessing the changes in the MMR and causes of death over this period. The protocol for this study and analysis of the causes of death has been published elsewhere.^17 18^ This paper describes changes in the MMR.

## Methods

### Interventions implemented

Findings of the 2007–2008 Zimbabwe Maternal and Perinatal Mortality Survey (ZMPMS) prompted the Zimbabwe Ministry of Health and Child Care (MoHCC) to implement a raft of interventions to reduce maternal mortality. A maternal and neonatal health roadmap was developed to address the direct and indirect causes of maternal mortality.^19^ Family planning services, prevention of mother-to-child transmission (PMTCT) of HIV services and community mobilisation for safe motherhood were scaled up through community health workers. Basic and comprehensive emergency obstetric and newborn care was rolled out in primary care and secondary/tertiary health facilities, respectively.^20–22^ The UK’s Royal College of Obstetricians and Gynaecologists and the Liverpool School of Tropical Medicine and Bristol University conducted training of trainers for 120 doctors and nurse–midwives who trained over 700 other doctors and nurses nationwide on the management of obstetrical and neonatal emergencies.^20^ Maternity waiting homes, which the MoHCC started establishing in the 1980s, were expanded in different districts, allowing women to stay at maternity facilities from the third trimester until delivery,^23–26^ increasing access to antenatal care and reducing home deliveries. The government developed guidelines for maternal and perinatal death surveillance and response system,^27^ and instituted maternal and perinatal death audits.^21^ Through a US$235 million health transition fund (2012–2015) and US$682 million health development fund (2016–2020), the government introduced free maternity services and doctors’ and nurses’ retention allowances in the rural provinces and supplied health facilities with essential commodities, among other initiatives, to improve maternity, neonatal and child healthcare.^11 12 28 29^

The 2007–2008 survey found that HIV was the major cause of maternal mortality, contributing 26% of maternal deaths; meaning that HIV interventions would significantly impact maternal mortality. In 2007–2008, Zimbabwe had high adult (15–49 years) HIV mortality.^30–33^ Antiretroviral therapy (ART) roll-out was in the early phases at this time.^34^ ART was available in only 5.2% (86/1643) of the health facilities by December 2007 and 17% (282/1643) by December 2008; mostly in secondary and tertiary hospitals that are less accessible to communities.^34^ In 2008 only 24% (148 144/596 965) of individuals needing ART received it and the need for ART was defined by a CD4 count below 350 cells/µ of blood at the time.^34 35^ However, HIV programmes received significant funding over the years, exceeding US$400 million annually.^36 37^ Consequently, ART was rolled out to 91% (1566/1722) of all health facilities in the country by 2017, and in 2019, 97% of adults with known HIV-positive status received ART.^38 39^ Adult HIV mortality substantially declined from an estimated 83 000 deaths in 2009 to 14 000 in 2018.^33 35 36^

In PMTCT, Zimbabwe rolled out WHO 2010 (‘Option A’) and 2013 (‘Option B+’) guidelines.^40 41^ Option ‘A’ was rolled out to 85% (1320/1560) MNCH facilities in 9 months and Option ‘B+’ to 88% (1385/1560) facilities in 5 months.^41^ All HIV-infected pregnant and breastfeeding women were initiated on lifelong ART, under the ‘Option B+’ guidelines, irrespective of disease stage. The combined impact of the ART and PMTCT programmes was that by 2019, 88% of adult women (15–49 years) living with HIV had known HIV-positive status, of which 98% were on ART.^39^ In 2018, 94% of HIV-positive pregnant women received ART for PMTCT.^36^ Without ART, pregnant women can die from AIDS-related complications including pneumonia, tuberculosis and meningitis. With a weakened immune system, HIV-infected pregnant women also have a higher risk of mortality from pregnancy-related sepsis, haemorrhage and other direct causes.^42–44^ The ART and PMTCT interventions should have contributed to the 91% reduction in HIV-related maternal mortality found in the causes of death analysis from this study.^18^

### Study design

A before-and-after analysis was performed using data from the two RAMOS conducted in 2007–2008 and 2018–2019. The surveys collected births and deaths among women in the reproductive ages (WRAs) 12–49 years, including maternal deaths, to analyse changes in Zimbabwe’s MMR in the context of the interventions described.

### Sampling method and sample size

The sampling method was designed for the first survey in 2007 and maintained in the second survey for comparability of the study findings. Two-stage cluster sampling was applied in the two surveys. In the first stage, the study population was clustered into the 10 provinces of the country, and one district was simple-randomly selected from each province. Two districts were selected from Harare province because of its large population and that several provinces refer complicated maternal cases to two central hospitals in the province. In the second stage, all births and deaths among WRAs 12–49 years from the selected districts were included in each survey.

Sample sizes of births required to calculate the MMR were calculated for each survey. In the first step, simple random samples were calculated using the Wald Test for a one-sample proportion (treating the MMR as a proportion).^45 46^ The recent MMRs from the Zimbabwe Demographic and Health Survey (ZDHS) of 2005–2006^47^ and 2015–2016 respectively were the expected proportions.^48^ Power of 80% and the z-value for two-sided 95% CI, continuity correction for normal approximation of the expected proportion and 2.5% error margin for the alternative hypothesis of MMR outside the 95% CI of the expected proportion were applied. In the second step, the random sample sizes were multiplied by the design effect (DE) to obtain the final sample sizes. The DE for the 2007–2008 survey was calculated from the pilot study and the DE for the 2018–2019 survey was calculated from the 2007–2008 survey (online supplemental table S2). The detailed procedure for calculating the sample size is also described in the study protocol.^17^ The two surveys required sample sizes of 45 000 and 71 500 births respectively.

### Study setting

**Figure 1:**
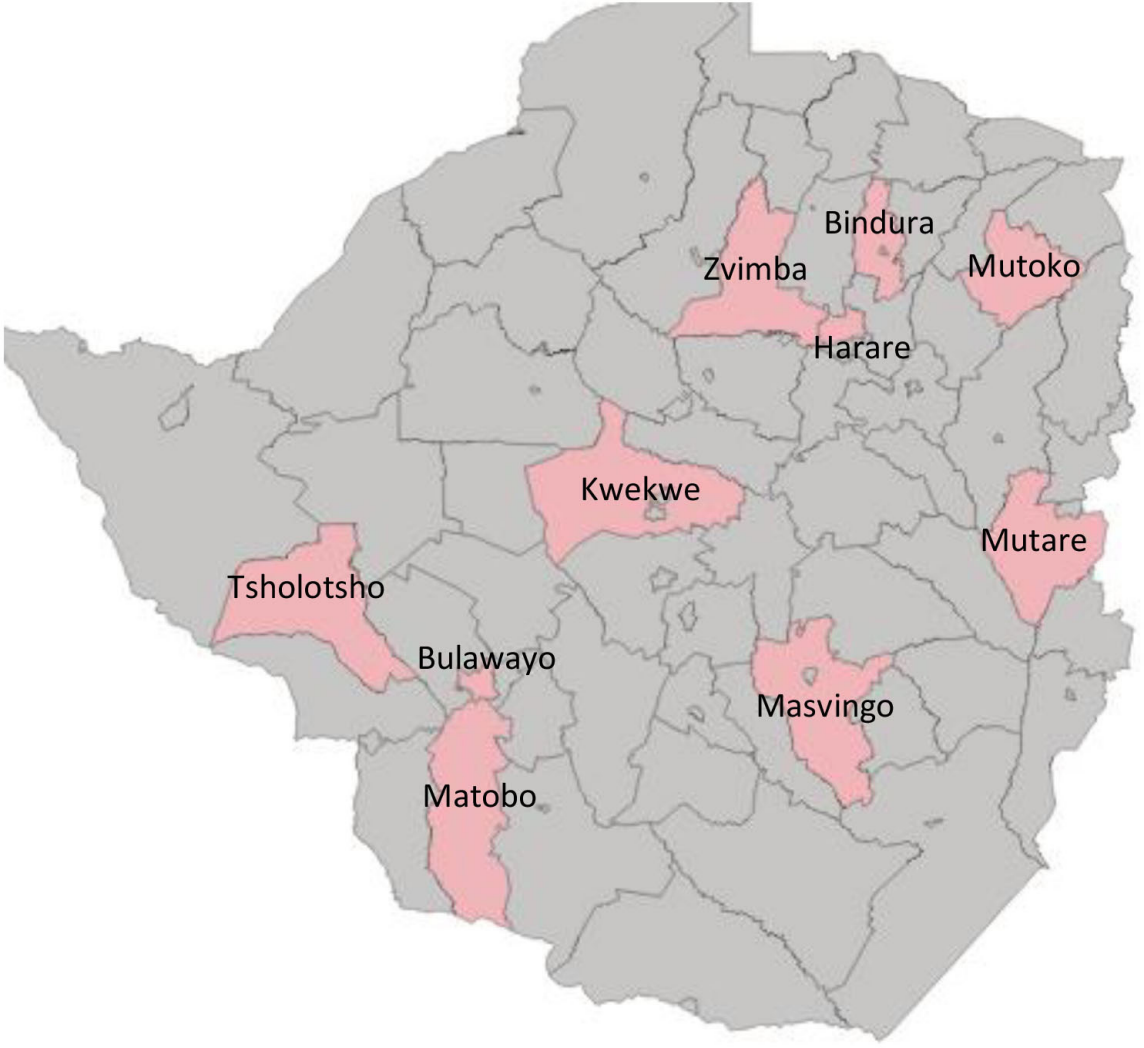
Map of study districts for Zimbabwe maternal and perinatal mortality study 2007–2008 and 2018–19.

Figure 1 shows the 11 study districts. Nkulumane (Bulawayo province), Western and South-Eastern districts (Harare province) are urban districts, while Mutare (Manicaland province), Bindura (Mashonaland East province) and Kwekwe (Midlands province) are semiurban districts and the rest are rural districts.

### Study variables

For each death (including pregnancy-related deaths) among WRAs, we collected location information (province, district and place of residence—urban or rural), age (in completed years), pregnancy status (pregnant or not) and cause of death (as stated on medical records and death certificates). For pregnancy-related deaths, we also collected information on parity, gestational age, antenatal care, pregnancy and delivery complications, referrals to other health institutions, delivery outcome and place of death (home or institutional).

### Data collection period

The first survey collected data for the period 1 May 2007 to 15 June 2008 and the second for the period 1 May 2018 to 15 June 2019. Data for the first survey were collected prospectively during the study period and data for the second survey were collected retrospectively from 1 May 2020 to 31 July 2020 and from 3 May 2021 to 20 July 2021.

### Data collection procedures

The 2007–2008 survey collected data from civil registration and vital statistics (CRVS) records at the government Registrar General (RG)’s offices, health facilities and the community. Study nurse–midwives trained on the study protocol and supervised by the investigators collected the data. They collected data on deaths of WRAs from RG’s offices and data for live births and pregnancy-related deaths in health facilities and the community for eligible women. Data for health facility deaths occurring during pregnancy or post-delivery were collected from medical records in the labour ward, theatre, high dependency and intensive care units, medical and surgical female wards, mortuaries and police posts. The survey was approved to collect identified data, hence, data collectors used the women’s personally identifying information (PII)—name, address, age and national identity numbers, to link individual women across these records.

Deliveries and deaths of WRAs occurring outside health institutions were enumerated in the community. Village health workers and village heads recorded them in study-provided register books. The research nurses followed up on every delivery and death recorded in the registers and interviewed the mothers (for births) and relatives (husband, mother, sister, aunty) for deaths, using study questionnaires. They collected additional data (dates when the death occurred, pregnancy status and signs and symptoms of sickness at death) for deaths of WRAs using a verbal autopsy (VA) form adapted from WHO.^49^ The data collectors used the women’s PII to cross-check and de-duplicate deaths identified in health facilities, the community and CRVS records. A group of six obstetrician–gynaecologists reviewed the data collection and VA forms for all pregnancy-related deaths, classified the deaths as maternal and non-maternal and assigned the causes of death.

In the 2018–2019 survey another group of nurse–midwives collected the data from the RG’s offices and health facility records (as in the 2007–2008 survey) and maternal death notification forms at the MoHCC’s district, provincial and national reproductive health offices. They cross-checked and de-duplicated the deaths using PII (as above). Live births data were collected from the MoHCC’s District Health Information System V.2 (DHIS2), a database system for health indicators.^50^ Nurses in the MNCH units recorded all institutional and home births presented at health facilities in birth registers. The nurses summarised the data on a standard monthly report form and submitted the reports to health information officers who entered the data into the DHIS2 database. Given the health system structure where deliveries occur in private and public health institutions, and health centres (rural and urban) refer complicated maternal cases to district hospitals, which refers to provincial hospitals, which also refers to central hospitals in Harare and Bulawayo, we counted the live births for women referred from the study districts to private, provincial and central hospitals, and added them to the DHIS2 births. We collected population data for WRAs for the study districts from the Zimbabwe National Statistics Agency (ZimStat).^51^

### Data verification, cleaning and classifying of the deaths

During the collection of 2018–2019 data, 2007–2008 deaths were verified in the CRVS records at the RG’s offices and health facility records (same sources as above). All questionnaires and VA forms for 2007–2008 deaths were reviewed by a new group of obstetrician–gynaecologists in 2020, the causes of death re-assigned and maternal deaths confirmed using the International Classification of Diseases V.10 manual for deaths during pregnancy, childbirth and puerperium (ICD-10 MM). The database for 2007–2008 deaths was cleaned for completeness and accurate data entry. The 2018–2019 data were collected in two rounds to ensure that all deaths in the source records were identified and correctly captured in the study.

### CRVS and health record systems in Zimbabwe

In Zimbabwe legislation regulates CRVS and medical records. The birth and death registration act mandates the registration and issuance of certificates for all births and deaths.^52^ The legislation mandates parents, health workers at institutions where the birth occurs or community leaders (for community births) to notify the RG’s office of the birth. Similarly, for persons who died at home, relatives or village heads are required by the law to notify the RG’s office, for the creation of a death record and issuance of a death certificate. Deaths that occur in health institutions get a medical death certificate signed by the doctor or nurse who attended the death. Home or community deaths attended to by the police are taken to hospitals where a doctor conducts a postmortem and issues another medical death certificate, stating the cause of death. The medical death certificates are deposited at the local RG’s office, where a record is created, and a civil death certificate is issued. The RG’s offices file birth and death records by date and year of registration and store them in secure record rooms. The public health act guides the recording, collection, storage, access, use, protection and confidentiality of health data.^53^

### Other studies reviewed

Reports presenting estimates of the MMR for Zimbabwe 2000–2019 were reviewed, including the ZDHS for 2000, 2005–2006, 2010–2011, 2015–2016^47 48 54^; the Multiple Cluster Indicator Survey (MICS) in 2014 and 2019^55 56^; population census in 2002 and 2012,^54 57^ the Maternal and Perinatal Death Surveillance Response in 2018 and 2019,^27^ and the United Nation’s Maternal Mortality Estimation Inter-Agency Group (MMEIG) MMR estimates for 2000 to 2017.^6 58–60^

### Definitions

WRAs are women aged 15–49 years, but children aged 12–14 years were included as some gave birth and died from maternal causes at this age. Pregnancy-related deaths were female deaths in which the woman was pregnant or within 42-days of termination of pregnancy, irrespective of the cause of death. Maternal deaths were deaths of women during pregnancy or within 42-days of pregnancy termination, irrespective of the duration and site of the pregnancy, from any cause related to or aggravated by the pregnancy or its management, but not from accidental or incidental causes.^61^

### Data analysis

We adjusted the number of maternal death for 2018–2019 for missed community deaths, using the 2007–2008 data as a standard, which comprehensively collected community deaths.^17 62^ The number of community deaths missed in 2018–2019 was estimated by equating the proportion of community to institutional deaths in the two surveys (online supplemental table S3).

We triangulated the total number of live births identified with the expected number of live births and estimated pregnancies obtained from ZimStat,^51^ to assess the completeness of the former. We calculated the expected number of live births by multiplying the 2018 populations of WRAs for each district with general fertility rates (GFRs) from the 2019 MICS survey,^55^ using the rural or urban GFR as applicable to each district. WHO recommends that when calculating MMRs using data from CRVS or health records, the live births must be corrected for missed births.^63^ As such, we calculated correction factors (expected/identified births), ranging from 1.0 to 1.3 (online supplemental table S4), and used them to correct the number of live births for each district for missed births.

We performed a before-and-after analysis of mortality using data from the two surveys calculating mortality incidence rates (IRs) of WRAs (number of deaths/1000 women) for each survey and the incidence rate ratios (IRRs) (mortality rate 2018–2019/mortality rate 2007–2008) and 95% CIs by district, age group and totally. Similarly, we computed the MMRs (number of maternal deaths/100 000 live births) and their IRRs for each district and totally. IRRs applied because the total person-years for each district cluster equalled the district population in 1 year. Half person-years were assigned to women who died during the year. We calculated the IRs using negative binomial models in Stata (V.17.0) immediate commands,^64^ treating the two surveys as cohorts, to use IRRs to estimate the magnitude of change in mortality levels between the two surveys. Stata immediate commands were employed because of the aggregate live births data in the IR denominators. Overall the IRs, MMRs and IRRs were weighted using the location variable (district) (online supplemental tables S5 and S6), to account for the clustering of the deaths within districts in the pooled samples. MMR 95% CIs were calculated using sampling errors (SEs) for the location variable. We calculated the SEs using the Jackknife repeated replication method used in the DHS^48^ (online supplemental table S5).

We also performed repeated measures’ one-way analysis of variance (ANOVA),^65^ for before-and-after comparison to confirm the statistical significance of the changes in the MMRs (online supplemental table S7). Using the WHO online calculator,^66^ we calculated the annual ARR for the country’s MMR from the study and the ARR needed to achieve the SDG target of 140 maternal deaths per 100 000 live births by 2030 from a 2015 ZDHS baseline MMR of 651 and 2019 MICS estimate of 462^55^ (online supplemental table S8).

### Patient and public involvement

There was no patient or public involvement in this study.

## Results

We identified 6188 deaths of WRAs in 2007–2008, of which 325 were pregnancy-related deaths and 296 were maternal deaths. The corresponding numbers for 2018–2019 were 1856, 137 and 130, respectively. In 2007–2008, community deaths constituted 50% (95/296) of the total maternal deaths. The unadjusted number of community deaths in 2018–2019 made up 19% (25/130) of the total maternal deaths identified. The number of maternal deaths for 2018–2019 adjusted for missed community deaths was 173 (table 1). The proportion of unadjusted maternal deaths (out of total deaths among WRAs) increased from 4.8% (296/6188) to 7.0% (130/1856) and 9.3% (173/1856) when adjusted for missed community deaths. The number of live births identified in the study constituted 81% of GFR-estimated live births and 83% of the expected pregnancies, justifying an average correction factor of 1.2 for the number of live births (online supplemental table S4). The unadjusted number of live births (for 2018–2019) was 67 225 and the adjusted number was 80 116.

**Table 1:** Maternal mortality ratio (MMR) in Zimbabwe 2007–2008 and 2018–2019 by district

District	2007–2008*					2018–2019†					IRR (95% CI)
	Unweighted live births	Unweighted maternal deaths	Weighted live births	Weighted maternal deaths	MMR (95% CI)	Unweighted live births	Unweighted maternal deaths‡	Weighted live births	Weighted maternal deaths	MMR (95% CI)	
Nkulumane§	4002	51	4387	62	1274 (950 to 1672)	5363	23.5	5748	27	448 (287 to 665)	0.34 (0.20 to 0.56)
Harare SE§	1911	12	1995	13	628 (325 to 1094)	2912	4.3	3022	‡	137 (37 to 351)	0.22 (0.05 to 0.72)
Harare W§	4958	30	5563	33	605 (409 to 863)	8072	16.0	8977	18	198 (113 to 322)	0.33 (0.17 to 0.62)
Mutare	7975	57	9666	71	715 (542 to 925)	14 784	29.0	18 129	35	196 (131 to 282)	0.27 (0.17 to 0.44)
Bindura	3016	27	3230	30	895 (591 to 1300)	7777	8.4	8613	9	103 (40 to 203)	0.11 (0.05 to 0.26)
Mutoko	3035	22	3252	24	725 (455 to 1095)	6150	19.8	6662	22	325 (199 to 502)	0.45 (0.23 to 0.86)
Zvimba	5863	25	6729	27	426 (267 to 629)	10 811	27.3	12 497	32	250 (165 to 363)	0.59 (0.33 to 1.1)
Chivi	3834	22	4186	24	574 (360 to 867)	6322	9.8	6863	10	158 (76 to 291)	0.28 (0.12 to 0.61)
Tsholotsho	2697	14	2867	15	519 (284 to 869)	4187	4.2	4418	‡	96 (26 to 244)	0.18 (0.04 to 0.59)
Matobo	1961	6	2049	6	306 (112 to 665)	3340	12.0	3485	13	359 (186 to 627)	1.2 (0.41 to 3.8)
Kwekwe	6327	30	7347	33	474 (320 to 676)	10 398	18.6	11 949	21	183 (110 to 285)	0.39 (0.21 to 0.71)
Total unweighted	45 579	296	51 270	337	649 (576 to 723)	80 116	172.9	90 363	196	215 (183 to 248)	0.33 (0.27 to 0.40)
Total weighted¶					657 (485 to 829)					217 (164 to 269)	0.33 (0.28 to 0.39)

*April 2007 to 15 May 2008.

†April 2018 to 15 May 2019.

‡Adjusted for missed community deaths.

§Nkulumane district in Bulawayo, and South-Eastern and Western districts in Harare are entirely urban districts.

¶Weighted by district location to account for clustering of deaths within districts (where mortality risk factors are similar) in the pooled sample.

IRR, incidence rate ratio.

Mortality among WRAs declined significantly in all districts. The smallest decline occurred in Matobo (55%) and the greatest in Kwekwe district (88%). Mortality also declined in all age groups; the greatest decline occurred in the 30–34 (83%) and 35–39 (81%) year age groups, and the smallest in the 15–19 year group (48%). Weighted by district, mortality among WRAs declined by 76%, from 11 to 3 women per 1000 (table 2).

**Table 2:** Mortality rate in women of reproductive ages 12–49 years in Zimbabwe 2007–2008 and 2018–2019 by district, age of women and whether the death was pregnancy-related or not

	2007–2008			2018–2019			IRR* (95% CI)
	Population†	Deaths	IR‡	Population†	Deaths	IR‡	
District							
Nkulumane	41 464	1479	35.7	42 985	397	9.2	0.26 (0.23 to 0.29)
Harare SE§	24 193	141	5.8	31 755	54	1.7	0.29 (0.21 to 0.40)
Harare W§	62 211	635	10.2	81 655	227	2.8	0.27 (0.23 to 0.32)
Mutare	123 954	974	7.9	148 616	367	2.5	0.31 (0.28 to 0.35)
Bindura	43 220	326	7.5	58 789	133	2.3	0.30 (0.24 to 0.37)
Mutoko	37 956	331	8.7	44 069	82	1.9	0.21 (0.17 to 0.27)
Zvimba	67 396	548	8.1	85 113	156	1.8	0.23 (0.19 to 0.27)
Chivi	47 866	264	5.5	49 311	114	2.3	0.42 (0.33 to 0.52)
Tsholotsho	34 533	357	10.3	31 363	79	2.5	0.24 (0.19 to 0.31)
Matobo	28 037	314	11.2	25 052	128	5.1	0.46 (0.37 to 0.56)
Kwekwe	89 516	819	9.1	105 468	119	1.1	0.12 (0.10 to 0.15)
Age-group							
12–14	77 544	82	1.1	87 675	26	0.3	0.28 (0.17 to 0.44)
15–19	133 104	228	1.7	132 452	117	0.9	0.52 (0.41 to 0.65)
20–24	115 701	589	5.1	115 236	159	1.4	0.27 (0.23 to 0.32)
25–29	89 201	1090	12.2	104 593	194	1.9	0.15 (0.13 to 0.18)
30–34	63 110	1358	21.5	90 122	321	3.6	0.17 (0.15 to 0.19)
35–39	47 467	1278	26.9	73 296	383	5.2	0.19 (0.17 to 0.22)
40–44	41 202	794	19.3	60 250	348	5.8	0.30 (0.26 to 0.34)
45–49	33 015	769	23.3	40 551	308	7.6	0.33 (0.28 to 0.37)
Pregnancy-related death							
Yes	45 579	325	7.1	59 576	137	2.3	0.32 (0.26 to 0.39)
No	554 765	5863	10.6	644 600	1719	2.7	0.25 (0.24 to 0.27)
Total unweighted	600 344	6188	10.3	704 176	1856	2.6	0.26 (0.24 to 0.27)
Total weighted**	681 634	7176	10.5	803 614	2145	2.7	0.25 (0.24 to 0.27)

*Weighted by district location to account for clustering of deaths within districts (where mortality risk factors are similar) in the pooled sample.

†Year 2007 and 2018 population projections were from Zimbabwe’s Statistics Department (ZimStats).

‡Mortality rate obtained using the formula: (number died / population) × 1000.

§Harare South-Eastern and Harare Western Districts.

IR, incidence rate; IRR, incidence rate ratio.

MMRs declined in 9 of the 11 districts; the smallest decline occurring in Mutoko (41%) and the greatest in Bindura district (89%). The weighted overall MMR declined by 67%, from 657 (95% CI: 485 to 829) to 217 (95% CI: 164 to 269) per 100 000 live births (table 1), at an annual ARR of 10.1%. The annual ARR required to reach the 2030 target of 140 per 100 000, calculated using the 2015 ZDHS baseline MMR of 651, was 10.2%. The repeated measures ANOVA showed statistical significance in the decline in MMR between the two study periods (p<0.05) (online supplemental table S7).

## Discussion

**Figure 2:**
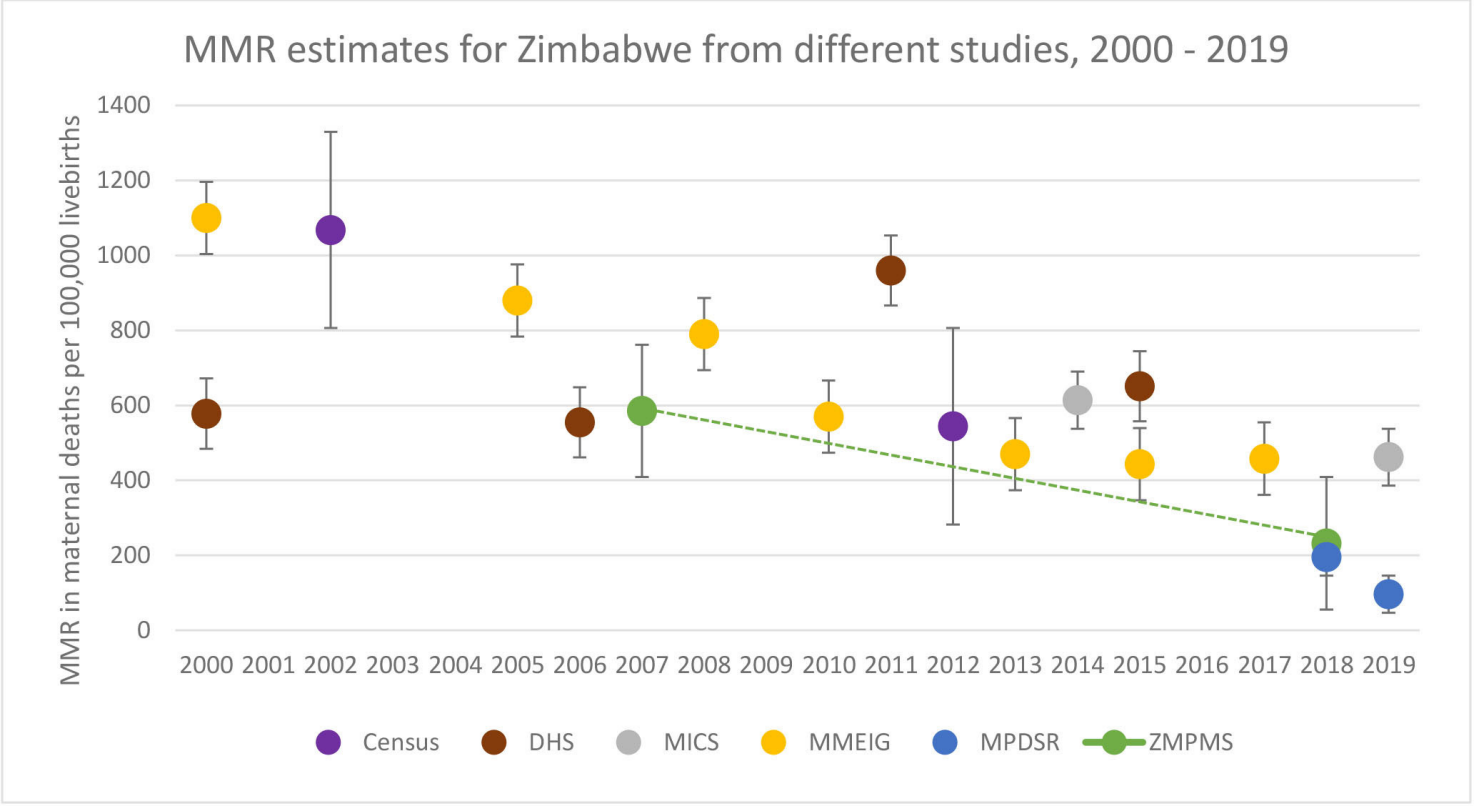
Zimbabwe MMR estimates from different data sources, 2002–2019. Census, Zimbabwe population census; DHS, Demographic and Health Survey; MICS, Multiple Cluster Indicator Survey; MMEIG, Maternal Mortality Estimation Inter-Agency Group; MMR, maternal mortality ratio; MPDSR, Maternal and Perinatal Death Surveillance and Response; ZMPMS, Zimbabwe Maternal and Perinatal Mortality Survey.

We found a significant decline in Zimbabwe’s MMR from 657 (95% CI: 485 to 829) in 2007–2008 to 217 (95% CI: 164 to 269) in 2018–2019, which is supported by other data sources (figure 2) and accompanied by a decline in overall reproductive age mortality. MMEIG estimated a decline from 790 in 2008 to 458 in 2017 at an annual ARR of 6.1%. The ZDHS estimated a decline from 960 in 2010 to 651 in 2015 at an ARR of 7.8%.

The decline in Zimbabwe’s MMR in this study translated to an annual ARR of 10.1%, against a target of 10.2% based on the 2015 ZDHS baseline MMR of 651, and a target of 10.9% based on the MICS MMR estimate of 462 in 2019 (online supplemental table S8).^55 66^ The targets are the ARRs required to achieve the SDG 3.1 supplementary target of individual countries reducing their MMRs to no more than 140 maternal deaths per 100 000 live births by 2030.^6^ Similar MMR declines have been recorded in other countries. Egypt’s MMR declined from 174 in 1992–1993 to 94 in 2000, at an annual ARR of 7.7%,^67^ and South Africa’s from 191 in 2007 to 101 in 2015, at an annual ARR of 8.0%.^68^

HIV interventions implemented between 2008 and 2018 could have contributed to the decline in Zimbabwe’s MMR. Analysis of the changes in causes of death from this study showed that HIV mortality decreased by 81% in WRAs and by 91% in pregnant women.^18^ The interventions against the direct causes of maternal mortality must have also contributed to the decline in the MMR, as the cause of death analysis showed a 61% decrease in pregnancy-related deaths due to direct causes.^18^

The 2018–2019 MMR estimate of 217 (95% CI: 164 to 269) is considerably lower than MICS’ 462 (95% CI: 288 to 538) and MMEIG’s 458 (95% CI: 360 to 577) estimates which used different methods. MICS used the sisterhood method (asking all adult respondents about the deaths of their sisters) to identify pregnancy-related deaths in a household survey that sampled enumeration areas (survey clusters) from the country’s provinces and estimated a 7-year (2013 to 2019) MMR.^69 70 70–72 72^ MMEIG used country data from different sources (CRVS, population-based sisterhood surveys, confidential enquiries into maternal deaths, RAMOS and any other sources that have clear data collection methods), along with robust statistical modelling.^6 7 69 73^

The SDG 3.1 and Ending Preventable Maternal Mortality supplementary targets are broad goals and statements of intent, set in full recognition of the absence of precise methods of measuring MMRs in different countries and that country targets depend on the baseline MMRs used. Zimbabwe’s estimates are not exempt from these nuances. Regardless, Zimbabwe’s MMR cannot continue declining at the annual ARR of 2009–2019 until 2030, for several reasons. Stabilisation of the economy from 2009 onwards from the 2000 to 2008 economic crisis led to stabilising of the health system and reduction of maternal mortality from the high levels of 2007–2008.^74^ However, health system constraints have resurfaced due to the rebounded economic challenges.^16^ The causes of death analysis showed a huge decline in deaths from indirect causes—mainly HIV deaths, due to increased ART coverage.^18^ However, the reduction in HIV mortality is flattening. At the same time, the causes of death analysis showed that mortality from direct causes is still three times higher than from indirect causes.^18^ Thus, Zimbabwe is unlikely to sustain the ARR of the 2009–2019 period.

### Strengths and weaknesses of the study

The strengths of our study are that it produced single-year MMRs while other studies estimated MMRs for a 5–7 year period. Our study includes a before-and-after analysis which allows us to report the ARR required to monitor the SDG 3.1 supplementary targets. Our study identified more maternal deaths, with causes of death information, compared with the sisterhood method studies.^48 55^ Trained obstetrician–gynaecologists classified the deaths into maternal and non-maternal deaths using the ICD-10 MM manual; therefore, misclassification of deaths may be low. Our study methods offer alternative approaches to assessing maternal mortality levels in limited-resource settings using CRVS and health records. Regular use of this method will strengthen these records, as documentation and classification of deaths and storage of records are interrogated during the data collection. The comprehensive data collected through this method enables the analysis of the causes of death to complement the MMR estimates. The sampling method used in 2007–2008 has some limitations, but the results can be generalised to the country since the district cluster samples followed the health system structure and geographically represented the country’s population. The sample sizes were powered to measure the MMR within acceptable error margins.

The limitations of our study are the use of different data sources for births and community deaths in the two surveys. Whereas the first survey enumerated births and deaths prospectively in the health facilities and community, the second survey used secondary data from CRVS and health records which would miss community births and deaths not registered in these data systems. To mitigate this, we statistically adjusted the births and maternal deaths data for 2018–2019, an approach which has its limitations. Notwithstanding, the adjustment for live births was validated. The triangulating data suggested undercounting of the live births by a consistent 20% (online supplemental table S4).

The adjustment of the number of maternal deaths in 2018–2019 would have a positive bias. The ZDHS reported an increase in institutional deliveries from 65% (57% rural and 85% urban) in 2010/2011 to 72% in 2015 (68% rural and 81% urban) and MICS reported 86% (82% rural and 94% urban) in 2019.^48^ Increase in institutional deliveries reduces pregnancy-related community deaths.^3 6 68 73^ Thus, by standardising the ratio of community to institutional deaths for 2018–2019 to the 2007–2008 ratio, the adjustment could inflate the 2018–19 maternal deaths and the MMR estimate.

## Conclusions

Between 2007–2008 and 2018–2019, Zimbabwe’s MMR declined from 657 to 217 alongside a decline in reproductive age mortality. The decline may be a result of the reduction in HIV mortality due to increased ART coverage and the impact of targeted MNCH interventions. However, since the reduction in HIV mortality may be flattening and mortality due to direct causes is still high, Zimbabwe may not sustain the annual ARR of 2009–2019. Therefore, we recommend scaling up interventions to reduce deaths due to direct maternal causes while sustaining interventions against HIV and other indirect causes.

## Data Availability

Data are available upon reasonable request.
