# Supplementary material for: Maternal mortality decline in Zimbabwe, 2007/2008 to 2018/2019: findings from mortality surveys using civil registration, vital statistics and health system data
Source: BMJ Glob Health. 2022 Aug 4;7(8):e009465. doi: 10.1136/bmjgh-2022-009465 (PMC9358939; doi:10.1136/bmjgh-2022-009465)
Supplement: Supplementary data [file bmjgh-2022-009465supp001.pdf]

Supplementary online document

Table S1: Background to Zimbabwe's maternal mortality ratio (MMR) changes, 2007-08 to 2018-19.

Zimbabwe experienced a severe socio-economic crisis that started in the late 1990s and reached its peak in 2008. The crisis caused hyperinflation and erosion of revenues, leading to government failure to adequately fund essential services, including health. Zimbabwe government's health strategies and numerous health sector assessments acknowledge this crisis.[1-4] The crisis caused deterioration of the health system to near-collapse, such that by 2009, about 80% of health facilities offering maternal, neonatal and child health services lacked essential commodities.[5] Skilled and experienced health workers deserted the health sector, such that in 2010, only 34% of doctors, 66% of nurses, 28% of pharmacists, 23% of environmental health officers and 45% of health services' administrator positions were staffed.[4] As the effects of the crisis continued, the health system scored 42% on general service availability, 69% on health infrastructure availability, 36% on core health workforce availability and 22% on service utilisation in a 2015 assessment.[6]

Zimbabwe being one of Southern Africa's high HIV burden countries, the HIV epidemic was taking its toll on the country in 2007-2008.[7-10] The roll-out of antiretroviral treatment (ART) had started in 2004 but was slow and was still in the early scaling-up phase by 2007-2008.[11] ART services were rolled out to only 5.2% (86 out of 1,643) of health facilities country-wide by December 2007 – mostly in provincial and tertiary hospitals, which are few and less accessible to communities.[12] In December 2008, 17% (282 out of 1643) health facilities offered ART services country-wide, and 24% (148,144/596,965) HIV-positive people were on ART (the need for ART defined by a CD4 count below 350 cells per microlitre (cells/µL) of blood).[12,13] In the same period, a debilitating cholera epidemic broke out in all provinces of the country from July to November 2008.[14]

In 2007-2008, the Ministry of Health commissioned the first of the two maternal mortality surveys included in this study. Preliminary findings of this survey reported an MMR of 725 maternal deaths per 100 000 livebirths, HIV complications being the leading cause, contributing to 26% of the deaths.[15] Concern arose locally and internationally about the findings. Consequently, the Ministry of Health and its partners instituted a raft of measures to address the maternal health situation. A maternal and neonatal health roadmap was developed to promote the four pillars of Safe Motherhood;[16] namely: (i) family planning and antenatal care, (ii) clean and safe delivery for the mother and newborn, (iii) essential obstetric and neonatal care – including obstetric and neonatal first aid, and (iv) basic emergency obstetric and neonatal care (BEmONC) and comprehensive obstetric and neonatal care (CEmONC). The strategies were (i) to train healthcare workers in these interventions, (ii) roll out BEmONC in primary care facilities and CEmONC in secondary and tertiary facilities, (iii) integrate family planning and prevention of mother-to-child transmission (PMTCT) of HIV services in Maternal, Newborn and Child Health (MNCH), and (iv) community mobilisation.

Following the crisis of 2007-2008, a government of national unity was formed in 2009, which began to reverse the socio-economic crisis.[17,18] Donor funding increased to support the country's socio-economic recovery, targeting strategic sectors such as health.[3] MNCH donors created two grants to revitalise the health system and improve MNCH services. The health transition fund (HTF) of US$ 235 million was implemented from 2012 to 2015, and the health development fund (HDF) of US$ 682 million, from 2016 to 2020.[17-19] European governments pooled the grants

through UNICEF. The HTF and HDF supported retention allowances for the health workforce (nurses and doctors) in rural areas and supply of essential medicines and commodities. Through a result-based financing (RBF) component, the grants funded operations and renovations in rural health facilities and community interventions. User fees were removed in rural health facilities. The availability of essential commodities such as antibiotics and blood for obstetric emergencies improved due to a dedicated monthly resupply program of emergency obstetric and neonatal care consumables.[5,20] Overall, fundamentals of Zimbabwe's health system began to improve.[21]

In the execution of the MNCH roadmap activities, the Ministry of Health rolled out trainings in the management of obstetric and neonatal emergencies nationwide, supported by the UK's Royal College of Obstetricians and Gynaecologists (RCOG) [22]. Trainers from the Liverpool School of Tropical Medicine (Making it Happen Project) and Bristol University (Alarm Project) facilitated the training of local trainers (120 doctors and nurse midwives became certified national trainers). In the end, more than 700 doctors and nurse midwives received the downstream training. During this time, the Ministry of Health established twelve new midwifery schools. The Ministry also resumed the training of advanced clinical officers, and expanded the training of nurse anaesthetists who were all recruited from rural district hospitals. Maternity waiting homes, which started in the 1980s, were expanded.[20,23-25] An additional 105 were established or renovated in rural health institutions, funded by the HTF and HDF, and other donors.

UNFPA, WHO, UNICEF and others supported the Ministry of Health to set up a Maternal and Perinatal Death Surveillance and Response (MPDSR) system.[26] Guidelines for maternal and perinatal death audits were developed.[27] The Ministry instituted the audit system, establishing audit committees at all health facilities providing maternity services and district, provincial and national levels of the Ministry. A surveillance system for reporting maternal deaths from health facilities to the Ministry's head office was established. Health facilities began to audit deaths, and complete and submit maternal death audit forms to the national level where the data were analysed. The national maternal and perinatal death audit committee audited some of the deaths reported by the sub-national committees. They gave feedback to the lower committees to improve the audits and management of maternal and neonatal cases at those levels. However, the surveillance system experienced challenges such as delayed and incomplete reporting but routine analysis of the data informed policies, scaling up of the interventions and resource allocation, while most audit recommendations would not be implemented because of limited resources.[28]

HIV programs also received significant funding through the crisis period, which exceeded US$ 400 million annually.[29,30] The Global Fund to Fight AIDS, Tuberculosis and Malaria and the United States President's Emergency Program for AIDS Relief (PEPFAR) were the main funders. Private donors and domestic funding from 3% of all taxable employee and business incomes complemented the donor funding.[30,31] With this funding, the country rolled out ART to 91% (1,566/1,722) health facilities by December 2017,[32] and about 97% of adults (15-49 years) with known HIV-positive status were on ART by 2019.[33]

Through the Elizabeth Glaser Pediatric AIDS Foundation (EGPAF), the Children's Investment Fund Foundation (CIFF) funded the roll-out of WHO 2010 ("Option A") and 2013 ("Option B+") guidelines for PMTCT by over US$ 100 million from 2011 to 2019. Under the "Option B+" guidelines, HIV-infected pregnant women were initiated on life-long ART irrespective of disease stage.[34,35] The ART and PMTCT programs had such an impact that by the year 2019,

88% of adult women (15-49 years) living with HIV had known status, of which 98% were on ART.[33] Consequently, a substantial decline in HIV-associated mortality in the adult populations was observed in 2009.[10]

Zimbabwe achieved these gains in the health sector because of targeted, evidence-based interventions implemented by the government and its partners. Maternal and perinatal health improved, despite a continued depression in other sectors of the country – farming, manufacturing, food security, employment, education, and others. The Government of Zimbabwe contributed to the funding of these interventions through its fiscus and the AIDS trust fund [3,36] but a significant proportion of the resources came from international donors.

Table S2: Design effect calculations for 2007-08 and 2018-19 surveys

District	Population (n_i)	Deaths (d_i)		Yi	Xi	R(i)	Ri	Ri-R	(Ri-R)^2
						R(i)=Yi/Xi	Ri=Kr-(k-1)R(i)		
Nkulumane	4387	62		46883	275	0.005871	0.013564577	0.00699438	4.89214E-05
Harare SE	1995	13		49275	324	0.006582	0.006448824	-0.0001214	1.47312E-08
Harare W	5563	33		45707	303	0.006639	0.005877185	-0.000693	4.80265E-07
Mutare	9666	71		41603	266	0.0064	0.008273054	0.00170286	2.89972E-06
Bindura	3230	30		48040	307	0.006393	0.00833751	0.00176731	3.1234E-06
Mutoko	3252	24		48018	313	0.00652	0.007070706	0.00050051	2.50509E-07
Zvimba	6729	27		44541	310	0.00695	0.002775653	-0.0037945	1.43986E-05
Chivi	4186	24		47084	313	0.00665	0.005776449	-0.0007937	6.30035E-07
Tsholotsho	2867	15		48403	322	0.006656	0.005715037	-0.0008552	7.31298E-07
Matobo	2049	6		49221	331	0.006719	0.005079101	-0.0014911	2.22337E-06
Kwekwe	7347	33		43923	303	0.006909	0.00318091	-0.0033893	1.14873E-05
**Total**	**51270**	**337**		**512698**	**3369**	**0.072289**		**-0.000173**	**0.000085160500**
k=11	R =	0.006570197						SE^2 =	7.74186E-07
CIs for MMR	0.004846	0.006570197	0.008295					SE (CDS)	0.000879879
100000	484.5635	657.0196581	829.4759					S^2(SRS)	0.005776361
								SE^2(SRS)	1.26733E-07
							**DE (2007-08)**		**6.108790912**

District	Population (n_i)	Deaths (d_i)		Yi	Xi	R(i)	Ri	Ri-R	(Ri-R)^2
						R(i)=Yi/Xi	Ri=Kr-(k-1)R(i)		
Nkulumane	5748	27		84615	169	0.001992	0.00391621	0.00174888	3.05858E-06
Harare SE	3022	4		87341	192	0.002195	0.001886315	-0.000281	7.89689E-08
Harare W	8977	18		81386	178	0.00219	0.001943584	-0.0002237	5.00621E-08
Mutare	18129	35		72234	161	0.002229	0.001553225	-0.0006141	3.77125E-07
Bindura	8613	9		81751	187	0.002287	0.000969152	-0.0011982	1.43563E-06
Mutoko	6662	22		83702	173	0.002073	0.003114492	0.00094716	8.97117E-07
Zvimba	12497	32		77866	163	0.002099	0.002848688	0.00068136	4.64249E-07
Chivi	6863	10		83500	185	0.002221	0.001627148	-0.0005402	2.91796E-07
Tsholotsho	4418	4		85945	192	0.002229	0.001554031	-0.0006133	3.76135E-07
Matobo	3485	13		86878	183	0.002106	0.002782394	0.00061506	3.78304E-07

Kwekwe	11949	21		78414	175	0.002232	0.001518568	-0.0006488	4.20891E-07
**Total**	**90363**	**195.8**	**0.00217**	**903631**	**1958**	**0.023853**		**-0.000127**	**0.000007828862**
k=11	R =	0.00216733						SE^2 =	7.11715E-08
CIs for MMR	0.001644	0.00216733	0.00269					SE (CDS)	0.00026678
100000	164.4441	216.732964	269.0218					SE^2(SRS)	4.03517E-08
								**DE (2018-19)**	**1.763779567**

Table S3: Calculations for adjustment of community maternal deaths missed in 2018-19, using the proportion of 2007-08 maternal deaths as a standard.

District	2007-08 Deaths			2018-19 Unadjusted Deaths			2018-19 Adjusted Deaths	
	Community (a)	Institutional (b)	Total	Community (d)	Institutional (e)	Total (t)	Missed Community Deaths (x)	Total Adjusted Deaths (T)
	a	b	-	d	e	t	x	T
Nkulumane-Bulawayo	12	39	51	1	18	19	5	23.5
Harare SE	0	12	12	0	4	4	0	4.0
Harare W	3	27	30	5	11	16	0	16.0
Mutare	13	44	57	7	22	29	0	29.0
Bindura	11	16	27	3	5	8	0	8.4
Mutoko	12	10	22	3	9	12	8	19.8
Zvimba	14	11	25	2	12	14	13	27.3
Chivi	13	9	22	1	4	5	5	9.8
Tsholotsho.	4	10	14	0	3	3	1	4.2
Matobo	4	2	6	0	4	4	8	12.0
Kwekwe	9	21	30	3	13	16	3	18.6
Total	95	201	296	25	105	130	43	172.6
NB: a to T denote the variables used in the model. The adjustment model formulas are: x = ae/b - d; T = t + ae/b - d								

Table S4: Adjustment of DHIS2 live births (2018-19) using correction factors from 2019 MICS' GFR and expected pregnancies data from ZimStat.

District	Population	General Fertility rate	Estimated births	Expected pregnancies 2019	DHIS2 Births	Proportion DHIS2 to GFR estimated livebirths	Proportion DHIS2 to expected pregnancies	Birth adjustment / correction factor	Adjusted births
Nkulumane-Bulawayo	42985	0.1006	4324	2775	5363	124%	193%	1.00	5363
Harare SE	31755	0.1006	3195	2057	2240	70%	109%	1.30	2912
Harare W	81655	0.1006	8214	5036	7081	86%	141%	1.14	8072
Mutare	148616	0.1006	14951	17112	13200	88%	77%	1.12	14784
Bindura	58789	0.1424	8372	6426	5982	71%	93%	1.30	7777
Mutoko	44069	0.1424	6275	5560	5348	85%	96%	1.15	6150
Zvimba	85113	0.1424	12120	10007	8316	69%	83%	1.30	10811
Chivi	49311	0.1424	7022	6318	4863	69%	77%	1.30	6322
Tsholotsho.	31363	0.1424	4466	4380	3221	72%	74%	1.30	4187
Matobo	25052	0.1424	3567	3574	2569	72%	72%	1.30	3340

Kwekwe	105468	0.1006	10610	11854	9042	85%	76%	1.15	10398
Total	704176		83117	75099	67225	81%	90%	1.21	80116
MICS - Multiple Indicator Cluster Survey; GFR - General Fertility Rate; ZimStat - Zimbabwe Statistical Agency									

Table S5: Weighting calculation for clustering-adjusted mortality rates among women of reproductive ages, 2007-08 and 2018-19, weighting by location (districts).

a) Weighting for population of women of reproductive ages, 2007-08

District	Population (n_i)	Deaths (d_i)	Proportion died (p_i)	Variance[1] (v_i)	Weight[2] (w_i)	Product (w_i*p_i)
Nkulumane	41464	1479	0.069066838	1.55E-06	6.45E+05	4.45E+04
Harare SE[1]	24193	141	0.040298428	1.60E-06	6.26E+05	2.52E+04
Harare W[1]	62211	635	0.103625243	1.49E-06	6.70E+05	6.94E+04
Mutare	123954	974	0.206470935	1.32E-06	7.57E+05	1.56E+05
Bindura	43220	326	0.071991818	1.55E-06	6.47E+05	4.66E+04
Mutoko	37956	331	0.063223541	1.56E-06	6.41E+05	4.05E+04
Zvimba	67396	548	0.112261929	1.48E-06	6.76E+05	7.59E+04
Chivi	47866	264	0.079730689	1.53E-06	6.52E+05	5.20E+04
Tsholotsho	34533	357	0.057521829	1.57E-06	6.37E+05	3.66E+04
Matobo	28037	314	0.046701402	1.59E-06	6.30E+05	2.94E+04
Kwekwe	89516	819	0.149107348	1.42E-06	7.06E+05	1.05E+05
Total	600346	6188	1.0000000000	0.00E+00	7285440	681634
**1** Variance = pi(1-pi)/n; 2 Weight = 1/vi; 3 Mortality rate = (∑wi*pi / ∑wi)*1000						

b) Weighting for deaths of women of reproductive ages, Incidence Rate (IR) and 95% Confidence Intervals (CI), 2007-08

District	Population (n_i)	Deaths (d_i)	Proportion died (p_i)	Variance[1] (v_i)	Weight[2] (w_i)	Product (w_i*p_i)
Nkulumane	41464	1479	0.239010989	1.23E-04	8.13E+03	1.94E+03
Harare SE[1]	24193	141	0.022786037	1.58E-04	6.33E+03	1.44E+02
Harare W[1]	62211	635	0.10261797	1.45E-04	6.90E+03	7.08E+02
Mutare	123954	974	0.157401422	1.36E-04	7.34E+03	1.16E+03
Bindura	43220	326	0.052682612	1.53E-04	6.53E+03	3.44E+02
Mutoko	37956	331	0.053490627	1.53E-04	6.54E+03	3.50E+02
Zvimba	67396	548	0.0885585	1.47E-04	6.79E+03	6.01E+02
Chivi	47866	264	0.042663219	1.55E-04	6.46E+03	2.76E+02
Tsholotsho	34533	357	0.057692308	1.52E-04	6.57E+03	3.79E+02
Matobo	28037	314	0.050743374	1.53E-04	6.52E+03	3.31E+02
Kwekwe	89516	819	0.132352941	1.40E-04	7.13E+03	9.44E+02
Total	600346	6188	1.0000000000	-3.70E-22	75244	7176
IR			0.01052734	1.03E+01	10.53	1.08E+01

c) Weighting for population of women of reproductive ages, 2018-19

District	Population (n_i)	Deaths (d_i)	Proportion died (p_i)	Variance[1] (v_i)	Weight[2] (w_i)	Product (w_i*p_i)
Nkulumane	42985	397	0.061042978	1.33E-06	7.50E+05	4.58E+04
Harare SE[1]	31755	54	0.04509526	1.36E-06	7.37E+05	3.33E+04
Harare W[1]	81655	227	0.115958226	1.26E-06	7.97E+05	9.24E+04
Mutare	148616	367	0.21104951	1.12E-06	8.93E+05	1.88E+05
Bindura	58789	133	0.083486231	1.30E-06	7.68E+05	6.41E+04
Mutoko	44069	82	0.062582366	1.33E-06	7.51E+05	4.70E+04
Zvimba	85113	156	0.12086893	1.25E-06	8.01E+05	9.68E+04
Chivi	49311	114	0.070026527	1.32E-06	7.57E+05	5.30E+04
Tsholotsho	31363	79	0.044538581	1.36E-06	7.37E+05	3.28E+04
Matobo	25052	128	0.035576333	1.37E-06	7.30E+05	2.60E+04
Kwekwe	105468	119	0.149775056	1.21E-06	8.28E+05	1.24E+05
Total	704176	1856	1.0000000000	0.00E+00	8549550	803614
[1] Variance (vi) = pi(1-pi)/n; [2] Weight (wi) = 1/vi; [3] Mortality rate = (∑wi*pi / ∑wi)*1000; [4] 95% CI = (p' +/- z*SQRT(p'(1-p')/n))*1000;						

d) Weighting for deaths of women of reproductive ages, Incidence Rate (IR) and 95% Confidence Intervals (CI), 2018-19

District	Population (n_i)	Deaths (d_i)	Proportion died (p_i)	Variance[1] (v_i)	Weight[2] (w_i)	Product (w_i*p_i)
Nkulumane	42985	397	0.213900862	4.24E-04	2.36E+03	5.05E+02
Harare SE[1]	31755	54	0.029094828	5.23E-04	1.91E+03	5.56E+01
Harare W[1]	81655	227	0.122306034	4.73E-04	2.11E+03	2.59E+02
Mutare	148616	367	0.197737069	4.32E-04	2.31E+03	4.57E+02
Bindura	58789	133	0.071659483	5.00E-04	2.00E+03	1.43E+02
Mutoko	44069	82	0.044181034	5.15E-04	1.94E+03	8.58E+01
Zvimba	85113	156	0.084051724	4.94E-04	2.03E+03	1.70E+02
Chivi	49311	114	0.061422414	5.06E-04	1.98E+03	1.21E+02
Tsholotsho	31363	79	0.042564655	5.16E-04	1.94E+03	8.25E+01
Matobo	25052	128	0.068965517	5.02E-04	1.99E+03	1.37E+02
Kwekwe	105468	119	0.064116379	5.04E-04	1.98E+03	1.27E+02
Total	704176	1856	1.0000000000	0.00E+00	22561	2145
IR, 95% CI			0.00266883	2.56E+00	2.67	2.78E+00

Table S6: Weighting calculations for clustering-adjusted maternal mortality ratio (MMR) among women of reproductive ages, 2007-08 and 2018-19.

a) Weighting for live births, 2007-08

District	Population (n_i)	Deaths (d_i)	Population proportion (p_i)	Variance[1] (v_i)	Weight[2] (w_i)	Product (w_i*p_i)
Nkulumane	4002	51	0.087804	2.00E-05	49966	4387

Harare SE	1911	12	0.041927	2.10E-05	47574	1995
Harare W	4958	30	0.108778	1.96E-05	51142	5563
Mutare	7975	57	0.174971	1.81E-05	55245	9666
Bindura	3016	27	0.066171	2.05E-05	48809	3230
Mutoko	3035	22	0.066588	2.05E-05	48831	3252
Zvimba	5863	25	0.128634	1.91E-05	52308	6729
Chivi	3834	22	0.084118	2.01E-05	49765	4186
Tsholotsho	2697	14	0.059172	2.06E-05	48446	2867
Matobo	1961	6	0.043024	2.10E-05	47628	2049
Kwekwe	6327	30	0.138814	1.89E-05	52926	7347
Total	45579	296	1.000000	0.00E+00	552639	51270
[1] Variance (vi) = pi(1-pi)/n; [2] Weight (wi) = 1/vi; [3] Mortality rate = (∑wi*pi / ∑wi)*1000; [4] 95% CI = (p' +/- z*SQRT(p'(1-p')/n))*1000;						

b) Weighting for maternal deaths, unweighted and weighted MMR, and 95% CIs, 2007-08

District	Population (n_i)	Deaths (d_i)	Proportion died (p_i)	Variance[1] (v_i)	Weight[2] (w_i)	Product (w_i*p_i)
Nkulumane	4002	51	0.172297	2.80E-03	358	62
Harare SE	1911	12	0.040541	3.24E-03	309	13
Harare W	4958	30	0.101351	3.04E-03	329	33
Mutare	7975	57	0.192568	2.73E-03	367	71
Bindura	3016	27	0.091216	3.07E-03	326	30
Mutoko	3035	22	0.074324	3.13E-03	320	24
Zvimba	5863	25	0.084459	3.09E-03	323	27
Chivi	3834	22	0.074324	3.13E-03	320	24
Tsholotsho	2697	14	0.047297	3.22E-03	311	15
Matobo	1961	6	0.020270	3.31E-03	302	6
Kwekwe	6327	30	0.101351	3.04E-03	329	33
Total	45579	296	1.000000	0.00E+00	3593	337
Unweighted MMR, 95% CI		0.0064942	0.00649422	5.7568E+02	649.42	7.232E+02
Weighted MMR, 95% CI			0.00657020	5.8709E+02	657.02	7.270E+02

c) Weighting for live births, 2018-19

District	Population (n_i)	Deaths (d_i)	Population proportion (p_i)	Variance[1] (v_i)	Weight[2] (w_i)	Product (w_i*p_i)
Nkulumane	5363	23.5	0.066940	1.16E-05	85864	5748
Harare SE	2912	4.0	0.036347	1.20E-05	83138	3022
Harare W	8072	16.0	0.100758	1.12E-05	89093	8977
Mutare	14784	29.0	0.184532	1.02E-05	98246	18129
Bindura	7777	8.4	0.097067	1.13E-05	88729	8613
Mutoko	6150	19.8	0.076766	1.15E-05	86778	6662
Zvimba	10811	27.3	0.134939	1.08E-05	92613	12497
Chivi	6322	9.8	0.078909	1.15E-05	86980	6863
Tsholotsho	4187	4.2	0.052265	1.18E-05	84534	4418

Matobo	3340	12.0	0.041686	1.20E-05	83601	3485
Kwekwe	10398	18.6	0.129790	1.09E-05	92065	11949
Total	80116	172.6	1.000000	0.00E+00	971641	90363

d) Weighting for maternal deaths, unweighted and weighted MMRs, and 95% CIs, 2018-19

District	Population (n_i)	Deaths (d_i)	Proportion died (p_i)	Variance[1] (v_i)	Weight[2] (w_i)	Product (w_i*p_i)
Nkulumane	5363	23.5	0.136377	5.00E-03	200	27
Harare SE	2912	4.0	0.023175	5.66E-03	177	4
Harare W	8072	16.0	0.092701	5.26E-03	190	18
Mutare	14784	29.0	0.168021	4.82E-03	207	35
Bindura	7777	8.4	0.048885	5.51E-03	181	9
Mutoko	6150	19.8	0.114718	5.13E-03	195	22
Zvimba	10811	27.3	0.158013	4.88E-03	205	32
Chivi	6322	9.8	0.056651	5.47E-03	183	10
Tsholotsho	4187	4.2	0.024334	5.65E-03	177	4
Matobo	3340	12.0	0.069526	5.39E-03	185	13
Kwekwe	10398	18.6	0.107599	5.17E-03	193	21
Total	80116	172.6	1.000000	0.00E+00	2094	196
Unweighted MMR, 95% CI		0.0021543	0.00215435	1.8333E+02	215.43	2.475E+02
Weighted MMR, 95% CI			0.00216733	1.8641E+02	216.73	2.471E+02

Table S7: ANOVA Tables[1] for repeated measures, before-and-after comparison of 2008-07 and 2018-19 MMRs, Zimbabwe RAMOS.

Given that the second survey used the same district clusters as the first survey, we performed additional statistical comparison using the repeated-measures method of One-Way ANOVA, using the online calculator available at https://www.socscistatistics.com/tests/anova/default2.aspx. The assumptions of the analysis are that the interventions and contextual factors of the MMR changes before 2007-08 and to 2018-19 measurement points are two treatments and comparison of the MMRs is comparison of two treatment effects. The data are fitted to a one-way ANOVA model because one factor, location, was available in the data for analysis.

Below are the results of the analysis in three tables, as guided by Ardelean's paper.[2]

a) Values from MMR calculations

District	2007-08 MMR	2018-19 MMR

[1] Calculations performed using the ANOVA online calculator available at https://www.socscistatistics.com/tests/anova/default2.aspx (Accessed 31 Jan 2022)
[2] MATEC Web of Conferences: Available at: chrome-extension://efaidnbmnnnibpcajpcglclefindmkaj/viewer.html?pdfurl=https%3A%2F%2Fwww.matec-conferences.org%2Farticles%2Fmatecconf%2Fpdf%2F2017%2F40%2Fmatecconf_imtoradea2017_04008.pdf&clen=413721&chunk=true (Accessed 31 Jan 2022).

Nkulumane	0.012744	0.004363
Harare South Eastern	0.006279	0.001920
Harare Western	0.006051	0.002260
Mutare	0.007147	0.002197
Bindura	0.008952	0.001404
Mutoko	0.007249	0.003871
Zvimba	0.004264	0.003283
Chivi	0.005738	0.001995
Tsholotsho	0.005191	0.001304
Matobo	0.003060	0.004671
Kwekwe	0.004742	0.002057
**Mean**	**0.0065000**	**0.0027000**

b) Summary of ANOVA results for the two periods

Groups	Count	Sum	STD Deviation	Variance
2008-08	11	0.0005	0.0026	
2018-19	11	0.0001	0.0012	
**Total**	**22**	**0.0006**	**0.0028**	

c) ANOVA results

Source of information	***SS***	***df***	***MS***	***P-value***	***F Critical***
Between-treatments	0.0001	1	0.0001	0.000263	F = 19.54935
Within-treatments	0.0001	20	0		
Total	0.0002	21			

Conclusion: The *f*-ratio value is 19.54935. The *p*-value is .000263. The result is significant at $p < .05$. Hence, the MMRs for the two study years are different.

Table S8: Calculations for Actual ARR and Required ARR to achieve the 2030 target of 140 per 100,000 from 2015 baseline MMR, Target ARR based on the 2007-08 RAMOS, 2015 ZDHS and 2019 MICS MMR Results.

ARR Calculated	Baseline Year (t0)	End year (t1)	Time (t1-t0)	MMR0	MMR1	ARR
Achieved_1 (ZMPMS)	2008	2019	11	657	217	-10.1
Achieved_2 (MMEIG)	2008	2017	9	790	458	-6.1
Achieved_3 (ZDHS)	2010	2015	5	960	651	-7.8
Target_1 (ZMPMS)	2008	2030	22	657	140	-7.0
Target_2 (ZDHS)	2015	2030	15	651	140	-10.2
Target_3 (MICS)	2019	2030	11	462	140	-10.9

Table S9: Zimbabwe Maternal and Perinatal Mortality Study (ZMPMS) Members 2007-08 and 2018-19

ZMPMS 2007-8 Group
Stephen Munjanja
Thulani Magwali
Gwendoline Kandawasvika
Davidzoyashe Makosa
Maxwell Chirehwa

ZMPMS 2018-2019 Group
Reuben Musarandega
Stephen Munjanja
Michael Nyakura
Thulani Magwali
Gerald Madziyire

Eunice Tahuringana
Margaret Nyandoro
Aveneni Mangombe
Esther Ngaru
Tsitsi Magure
Nhamo Gona
Vongai Dondo
Ronald Mataya
Bothwell Guzha
Jonathan Kasule
Taurai Gunguwo
Sarah Manyame
Julius Chirengwa
Velda Mushangwe
Gwendoline Chimhini
Sunhurai Mukwambo
McMillan Parirenyatwa
Agnes Mahomva
Bernard Madzima
Davidzoyashe Makosa
Lucia Gondongwe
Chipo Chimamise
Winston Chirombe
Grace Chaora
Solwayo Ngwenya
Enesia Ziki
Mercy Gaza
Chipo Gwanzura
Admire Chikutiro
Rumbidzai Makoni
Grant Murewanhema

References

1. MOHCC. National Health Strategy For Zimbabwe 2016-2020. http://documents1.worldbank.org/curated/en/364731563173429622/pdf/The-National-Health-Strategy-Equity-and-Quality-in-Health-Leaving-No-One-Behind-for-Zimbabwe-2016-2020.pdf (accessed 27 April 2021).
2. MOHCC. National Health Strategy, 2009 – 2013. https://extranet.who.int/nutrition/gina/sites/default/filesstore/ZWE%202009%20The%20National%20Health%20Strategy%20for%20Zimbabwe.pdf (accessed 27 April 2021).
3. JICA. Data Collection Survey on Health Sector: Country Report Republic of Zimbabwe. *2012.* https://openjicareport.jica.go.jp/pdf/12085064.pdf ()2012 (accessed 27 April 2021).
4. USAID. Zimbabwe health system assessment 2010. https://www.hfgproject.org/wp-content/uploads/2015/02/Zimbabwe_Health_System_Assessment20101.pdf (accessed 24 April 2021).
5. UNICEF. Comparing facility and community level evidence on health services performance 2009 and 2010, Zimbabwe. https://www.tarsc.org/publications/documents/CMP%20and%20VMAHS%20rep2011.pdf (27 April 2021).
6. MOHCC. Zimbabwe Service Availability and Readiness Assessment 2015: Assessment Report. http://ncuwash.org/newfour/wp-content/uploads/2017/08/Zimbabwe-Service-Availability-and-Readiness-Assessment-Report.pdf (accessed 27 April 2021).
7. Gregson S, Gonese E, Hallett TB, et al. HIV decline in Zimbabwe due to reductions in risky sex? Evidence from a comprehensive epidemiological review. *International journal of epidemiology* 2010; **39**(5): 1311-23.
8. Mahomva A, Greby S, Dube S, et al. HIV prevalence and trends from data in Zimbabwe, 1997-2004. *Sex Transm Infect* 2006; **82 Suppl 1**: i42-7.
9. Hargrove JW, Humphrey JH, Mahomva A, et al. Declining HIV prevalence and incidence in perinatal women in Harare, Zimbabwe. *Epidemics* 2011; **3**(2): 88-94.
10. Gregson S, Mugurungi O, Eaton J, et al. Documenting and explaining the HIV decline in east Zimbabwe: the Manicaland General Population Cohort. *BMJ Open* 2017; **7**(10): e015898.
11. Apollo T, Takarinda K, Mugurungi O, Chakanyuka C, Simbini T, Harries AD. A report on the Zimbabwe Antiretroviral Therapy (ART) programme progress towards achieving MGD6 target 6B: achievement and challenges. *Cent Afr J Med* 2010; **56**(1-4): 12-4.
12. MOHCC. Zimbabwe OI/ART Programme Annual Report 2008. Ministry of Health and Child Care Zimbabwe, OI/ART Unit; Harare, 2009.

13. MOHCC. Zimbabwe national HIV estimates 2009. Ministry of Health and Child Care Zimbabwe; Harare, 2010.
14. Cuneo CN, Sollom R, Beyrer C. The Cholera Epidemic in Zimbabwe, 2008-2009: A Review and Critique of the Evidence. *Health Hum Rights* 2017; **19**(2): 249-64.
15. Munjanja SP, Nyandoro M, Magwali T. Zimbabwe Maternal and Perinatal Mortality Study 2007: Short Report. Ministry of Health and Child Welfare; Harare, 2007.
16. MOHCC. Zimbabwe Maternal and Neonatal Health Roadmap. Zimbabwe Ministry of Health and Child Care; Harare, 2007.
17. UNICEF. Independent evaluation of the Health Development Fund (HDF). https://www.unicef.org/zimbabwe/media/936/file/Independent%20evaluation%20of%20the%20Health%20Development%20Fund%20(HDF).pdf (accessed 7 May 2021).
18. D'Aquino L, Pyone T, Nigussie A, Salama P, Gwinji G, van den Broek N. Introducing a sector-wide pooled fund in a fragile context: mixed-methods evaluation of the health transition fund in Zimbabwe. *BMJ Open* 2019; **9**(6): e024516.
19. UNICEF. Independent evaluation of the Health Development Fund (HDF). https://www.unicef.org/zimbabwe/reports/independent-evaluation-health-development-fund-hdf (accessed 10 April 2021).
20. Crown Agents. Waiting homes – the initiative helping more mothers give birth safely in Zimbabwe. 7th April 2021 2021. https://www.crownagents.com/blog-post/waiting-mothers-homes-the-initiative-helping-more-mothers-give-birth-safely-in-zimbabwe/ (accessed 19 July 2021).
21. USAID. Assessment of opportunities to deliver oral PrEP for women through private sector health care: Zimbabwe research findings. https://www.prepwatch.org/wp-content/uploads/2017/09/Private-Health-Sector-Landscape-Zimbabwe.pdf (accessed 27 April 2021).
22. RCOG. Life Saving Skills Manual: Essential Obstetric and Newborn Care. 2nd ed. The Royal College of Obstetricians and Gynaecologist; Glasgow, 2007.
23. Tumwine JK, Dungare PS. Maternity waiting shelters and pregnancy outcome: experience from a rural area in Zimbabwe. *Ann Trop Paediatr* 1996; **16**(1): 55-9.
24. UNICEF. A 'Sheltering' Grace: The maternity waiting home giving hope to mothers in rural Zimbabwe. 10 May 2021 2021. https://www.unicef.org/zimbabwe/stories/sheltering-grace-maternity-waiting-home-giving-hope-mothers-rural-zimbabwe (accessed 19 July 2021 2021).
25. UNFPA. Maternity Waiting Homes: Promoting Institutional Delivery and Pregnant Women's Access to Skilled Care2012. https://zimbabwe.unfpa.org/sites/default/files/pub-pdf/maternitywaitinghomes.summary.pdf (accessed 19 July 2021).
26. MOHCC. Maternal and Perinatal Death Surveillance and Response Report. Ministry of Health and Child Care Zimbabwe: Harare, 2018.
27. MOHCC. Guidelines for Maternal and Perinatal Death Audits in Zimbabwe. Zimbabwe Ministry of Health and Child Care; Harare, 2013.
28. MCHIP. Assessment of Maternal and Perinatal Death Surveillance and Response Implementation in Zimbabwe. *Technical report* 2017. https://www.mcsprogram.org/resource/assessment-maternal-perinatal-death-surveillance-response-implementation-zimbabwe/ (accessed 7 April 2021).
29. MOHCC. Global AIDS Monitoring: Zimbabwe country progress report 2019. https://www.unaids.org/sites/default/files/country/documents/ZWE_2019_countryreport.pdf (accessed 7 May 2021).
30. UNAIDS. UNAIDS Data 2019. United Nations Joint Program on HIV and AIDS. https://www.unaids.org/sites/default/files/media_asset/2019-UNAIDS-data_en.pdf (accessed 19 July 2021).
31. Avert. Global information and education on HIV and AIDS: HIV AND AIDS IN ZIMBABWE. 24 August 2020 2021. https://www.avert.org/professionals/hiv-around-world/sub-saharan-africa/zimbabwe (accessed 19 July 2021).

32. ICAP. Zimbabwe Population-based HIV Impact Assessment (ZIMPHIA) 2015–2016. https://phia.icap.columbia.edu/zimbabwe-final-report/ (accessed 14 November 2020).
33. ICAP. Zimbabwe Population-based HIV Impact Assessment (ZIMPHIA) 2020. https://phia.icap.columbia.edu/zimbabwe-2020-summary-sheet/ (accessed 14 November 2020).
34. Musarandega R, Robinson J, Sen PD, et al. Using the critical path method to rollout and optimise new PMTCT guidelines to eliminate mother-to-child transmission of HIV in Zimbabwe: a descriptive analysis. *BMC Health Services Research* 2020; **20**(1).
35. MOHCC. Global AIDS response progress report 2018: Zimbabwe country progress report for reporting period January - December 2017. https://www.unaids.org/sites/default/files/country/documents/ZWE_2018_countryreport.pdf (accessed 14 May 2021).
36. Bhat N, Kilmarx PH, Dube F, Manenji A, Dube M, Magure T. Zimbabwe's national AIDS levy: A case study. *SAHARA J* 2016; **13**(1): 1-7.
